# Clinical validation of full genotyping CLART® HPV4S assay on SurePath and ThinPrep collected screening samples according to the international guidelines for human papillomavirus test requirements for cervical screening

**DOI:** 10.1186/s12885-020-06888-0

**Published:** 2020-05-06

**Authors:** Ditte Møller Ejegod, Camilla Lagheden, Ramya Bhatia, Helle Pedersen, Elia Alcañiz Boada, Karin Sundström, Javier Cortés, F. Xavier Bosch Josë, Kate Cuschieri, Joakim Dillner, Jesper Bonde

**Affiliations:** 1 Molecular Pathology Laboratory, Department of Pathology, Hvidovre Hospital, Copenhagen University Hospital, Kettegård Allé 30, 2650 Hvidovre, Denmark; 2 Department of Laboratory Medicine, Karolinska Institutet, and Karolinska University Laboratory, Karolinska University Hospital, Forskningsgatan, F56 14186 Stockholm, Sweden; 3 HPV Research Group, University of Edinburgh, Queen’s Medical Research Institute, 47 Little France Crescent, Edinburgh, EH16 4TJ Scotland, UK; 4 Spanish Society of Obstetrics and Gynecology, Palma, Spain; 5 Cancer Epidemiology Research Program, Catalan Institute of Oncology, Granvia de L’Hospitalet 199-203, Barcelona, Spain; 6 Scottish HPV Reference Laboratory, Department of Laboratory Medicine, Royal Infirmary of Edinburgh, 51 Little France Crescent, EH16 4SA Edinburgh, Scotland, UK

## Abstract

**Background:**

To ensure the highest quality of human papillomavirus (HPV) testing in primary cervical cancer screening, novel HPV assays must be evaluated in accordance with the international guidelines. Furthermore, HPV assay with genotyping capabilities are becoming increasingly important in triage of HPV positive women in primary HPV screening. Here we evaluate a full genotyping HPV assay intended for primary screening.

**Methods:**

The CLART® HPV4S (CLART4S) assay is a newly developed full-genotyping assay detecting 14 oncogenic (16, 18, 31, 33, 35, 39, 45, 51, 52, 56, 58, 59, 66, 68) and two non-oncogenic HPV genotypes (6, 11). It was evaluated using SurePath and ThinPrep screening samples collected from the Danish and Swedish cervical cancer screening programs, respectively. For calculation of sensitivity, 81 SurePath and 80 ThinPrep samples with confirmed ≥CIN2 were assessed. For clinical specificity analysis, 1184 SurePath and 1169 ThinPrep samples from women with <CIN2 histology were assessed. Sensitivity and specificity of the CLART4S assay was compared to an established reference test; the MGP-PCR (Modified General Primers GP5+/6+ with genotyping using Luminex). Inter and intra laboratory reproducibility of the assay was assessed using 540 SurePath and 520 ThinPrep samples, respectively. The genotype concordance between CLART4S and MGP-PCR was also assessed.

**Results:**

In SurePath samples, the sensitivity of CLART4S was 0.90 (MGP-PCR =0.93) and the specificity was 0.91 (MGP-PCR = 0.91); In ThinPrep samples the sensitivity of CLART4S was 0.98 (MGP-PCR = 1.00) and specificity was 0.94 (MGP-PCR =0.87). The CLART4S was shown to be non-inferior to that of MGP-PCR for both sensitivity (*p* = 0.002; *p* = 0.01) and specificity (*p* = 0.01; *p* = 0.00) in SurePath and ThinPrep samples, respectively. Intra-laboratory reproducibility and inter-laboratory agreement was met for both media types. The individual genotype concordance between CLART4S and MGP-PCR was good agreement for almost all 14 HPV genotypes in both media types.

**Conclusions:**

The CLART4S assay was proved non-inferior to the comparator assay MGP-PCR for both sensitivity and specificity using SurePath and ThinPrep cervical cancer screening samples from the Danish and Swedish screening programs, respectively. This is the first study to demonstrate clinical validation of a full-genotyping HPV assay conducted in parallel on both SurePath and ThinPrep collected samples.

## Background

Human Papillomavirus (HPV)-based cervical cancer screening is currently used in several countries including Netherlands, US, Denmark, Norway, Sweden, Spain and Australia, with several more countries planning for implementation. Compared to cytology, HPV based screening has superior clinical sensitivity and negative predictive value [1, 2]. Today, more than 200 molecular HPV assays are commercially available [3], and clinical validation remains pivotal to ensure screening-relevant assay performance. The 2009 international guidelines on HPV test validation defined the clinical performance criteria for novel HPV assays based on performance relative to that of Hybrid Capture 2 (HC2) or GP5+/6 + −PCR, which was both validated through randomized trials [4]. Additionally, the international guidelines also defined a set of inter- and intra-laboratory reproducibility requirements to ensure clinical routine performance.

A decade later, the 2009 validation criteria remain the highest level of validation yet updates on several pivotal points could be suggested. Firstly, HPV based screening will, for a period to be, run on liquid-based cytology (LBC) collection media, most notably SurePath and ThinPrep. This allows for HPV screening and subsequent cytology triage of HPV positive samples on one and the same specimen. Yet, these medias are different in chemical formulation, and most importantly, sample collection volume. Secondly, the defined comparator assays are more or less out-phased in clinical use or modified to the point where generating a strictly compliant reference panel for validation of new HPV assay has become an undue costly and complicated affair. Thirdly, the 2009 criteria do not embrace the technological development towards assays with genotyping, in that the criteria only assesses sensitivity and specificity performance on all HPV genotypes combined, not at individual genotype level. Yet, the current state-of-the-art screening algorithms from many countries acknowledges and utilizes genotyping of at least HPV16 and HPV18, with more genotypes assigned risk and specific management as new screening algorithms are implemented.

Multiple assays have been internationally validated and/or FDA approved for one but rarely both LBC collection media [5–14], with the BD Onclarity HPV test being the exception [15–17]. The lack of validation on both medias represents a clear challenge for introduction of HPV based screening. Consequently, simultaneous validation of HPV tests on the two market-leading cytology sample collection systems arguably offers a more comprehensive evaluation of novel assays.

The value of genotyping is based upon evidence that HPV genotypes have different oncogenic potential [18–23]. HPV16 and HPV18 contribute to approximately 70% of all cervical cancers; the five HPV genotypes HPV 31, 33, 45, 52 and 58 are associated with a further 19% of cervical cancers, whereas the remaining six oncogenic HPV genotypes HPV35, 39, 51, 56, 59 and 68 contribute 8–9% [18, 24]. Other HPV genotypes are only rarely involved in cervical carcinogenesis, with HPV 66 categorized as possibly oncogenic [25]. On top of this, the HPV genotype specific risk of Cervical Intraepithelial Neoplasia (CIN) 3 is also age dependent. HPV16 confers the largest risk in women below 30 years of age [26], whereas HPV16 in combination with 18, 31 and 33 together constitute the highest relative risk of disease in women above 30 years of age [26–29]. Data on the absolute risk of CIN by individual HPV genotypes and the fast evolution of cervical screening technology makes it increasingly relevant to consider HPV type specific risk-based screening algorithms [19, 30]. From a guideline perspective, risk stratification based on HPV16 and HPV18 is already incorporated into a number of national guidelines for triage of HPV positive screening samples [31], as standalone referral indication for colposcopy or as part of a combined outcome with cytology findings of atypical squamous cells of undetermined significance (ASCUS) or low-grade squamous intraepithelial lesion (LSIL) in certain settings [28, 30, 32, 33].

The CLART® HPV4S (CLART4S) microarray assay (GENOMICA SAU, Madrid, Spain) is intended as a primary HPV screening assay detecting individually 14 HPV genotypes (16, 18, 31, 33, 35, 39, 45, 51, 52, 56, 58, 59, 66 and 68). Additionally, the assay detects two non-oncogenic-HPV genotypes, HPV6 and 11.

Here, we assessed the clinical performance of the CLART4S assay relative to that of the comparator assay MGP-PCR (Modified General Primers GP5+/6+ with genotyping using Luminex (BioRad) assay) using the international guidelines for primary cervical cancer screening [4]. The CLART4S was evaluated in both SurePath and ThinPrep collected cervical cancer screening samples, from women aged 30–65 participating in the Danish and women aged 23–60 years participating in the Swedish cervical cancer screening programs, respectively.

## Methods

### Sample selection

#### SurePath cervical cancer screening samples

For the specificity analysis (the “no disease” *control* population), 1395 residual SurePath samples were collected from Danish women ≥30 years undergoing routine cervical cancer screening at Hvidovre Hospital, Denmark. Collection of the control panel was completed in October 2016. In total, 211 samples were excluded due to one of the following reasons 1) women with previous cytological diagnosis of ASCUS within the past 15 months; 2) a cytological diagnosis of more than ASCUS (>ASCUS) in the past 12 months; 3) previous cervical cancer or CIN in the previous 3 years; or 4) insufficient/incomplete histological follow-up in the Danish register or diagnosis of ≥CIN2 follow-up after baseline analysis, 5) laboratory processing and/or technical errors. The final control population incorporated 1184 samples (mean age 43.4, range 30–65).

For the sensitivity analysis (the “disease” *case* population) residual SurePath material from 411 consecutive, unselected samples were collected from Danish women undergoing screening between September and October 2012 at Hvidovre Hospital. The samples were derived from women with ≥ASCUS cytology. After collection, samples with insufficient material for testing were excluded and from the remainder women ≥30 years with confirmed ≥CIN2 histology were selected, yielding 57 samples in total. In June 2016, an additional 24 samples were selected from 70 consecutive ≥ASCUS samples using the same criteria. In total, the case population consisted of 81 samples from women ≥30 years of age with confirmed ≥CIN2 (mean age 40.3, range 30 to 73).

For assay reproducibility, 474 samples included in the control population were selected. In addition, 70 samples with ≥ASCUS cytology were collected from the routine cervical screening at Hvidovre hospital, to ensure compliance with the requirement for a 30% HPV positive rate within the reproducibility element [4]. Four samples were excluded due to technical invalidity in one of three runs for the reproducibility element. In total, DNA from 540 samples were included constituting 379 MGP-PCR negative and 161 MGP-PCR positive samples. An aliquot of extracted DNA from the 540 reproducibility samples was shipped to the HPV Research Group, University of Edinburgh, who performed the inter-laboratory agreement testing.

#### ThinPrep cervical cancer screening samples

For the specificity analysis (the “no disease” *control* population), all women between 01-jan-2013 and 31-dec-2015 undergoing routine cervical screening in Stockholm county, Sweden was included, in total 290,793 samples, of these 117,365 had sample residuals stored in the Clinical Cytology Biobank, Karolinska University Laboratory, Stockholm, Sweden. Subsequently all women with both the current and previous cytology classified as normal (*n* = 92,695) were identified. From these, a random set of 1169 samples with sufficient material was drawn (mean age 38.3, range 30–63).

For the sensitivity analysis (the “disease” case population), all women with ≥CIN2 diagnosed in routine cervical screening in Stockholm, Sweden, who had residual samples stored in the Clinical Cytology Biobank, Karolinska University Laboratory, Stockholm, Sweden, were identified from 01-jan-2013 to 31-dec-2015 (*n* = 4274). From here 80 consecutive samples from women ≥23 years of age with confirmed ≥CIN2 (mean age 34, range 23–60) was selected. Of these, 21 samples were derived from women < 30 and 59 samples derived from women ≥30 years of age.

For the reproducibility analysis, samples derived from women participating in primary HPV-based screening in Stockholm, stored in the Clinical Cytology Biobank, Karolinska University Laboratory, were identified starting from 1-sep-2014 (the start of primary HPV-screening above 30) to 30-nov-2014. The first 160 consecutive samples registered as HPV-positive and the first 360 consecutive samples registered as HPV-negative were included, for a total of 520 samples. An aliquot of extracted DNA from the 520 reproducibility samples was shipped to the HPV Research Group, University of Edinburgh, where inter-laboratory agreement testing was performed. In total 491 and 495 valid samples were included in the intra and inter-laboratory reproducibility element, respectively.

### DNA extraction

The MagNA Pure 96 platform (Roche diagnostics, Rotkreutz, Switzerland) with MagNA Pure LC total nucleic acid isolation kit (Roche Diagnostics) was used for both the SurePath and ThinPrep samples. For SurePath; 1 ml of material was preprocessed with heat treatment for 1 h at 56 °C with proteinase K, followed by 1 h at 90 °C to reverse formaldehyde-induced cross-linking prior to extraction. Extracted DNA was stored refrigerated prior to CLART4S and MGP-PCR testing. For ThinPrep, an aliquot of 100 μL from all included ThinPrep samples was extracted, and the resulting DNA was stored in − 20 °C prior to CLART 4S and (MGP-PCR) Luminex testing.

### GENOMICA CLART® HPV4S assay

GENOMICA CLART® HPV4S is a PCR-based microarray assay that targets the HPV L1 region, and detects 16 individual genotypes: HPV16, 18, 31, 33, 35, 39, 45, 51, 52, 56, 58, 59, 66, 68 and HPV6 and 11. The assay has two internal controls: one for PCR performance and one for sample sufficiency and assay performance. The internal control for PCR processing relies on amplification of a spiked CFTR plasmid, and is used to validate the individual PCR run, the internal control for human CFTR is used to validate sufficient human material in the sample. The assay is fully automated after PCR amplification using the autoclart®plus platform. In short, 5 μl aliquots of extracted DNA were used for the CLART HPV4S PCR amplification. Prior to visualization on low-density microarrays, the PCR products were denaturated at 95 °C for 10 min. Visualization and reporting of genotyping results were done automatically on the Clinical Array Reader (CAR®) as part of the automated autoclart®plus workflow. All samples with an invalid result (no human CFTR amplification detected or no spiked CFTR plasmid amplification detected) were retested once, and the second result was considered definitive. The CLART4S assay run-protocol was independent of sample media. As part of the validation, a posteriori optimization of genotype specific cut off values was conducted against detection of ≥CIN2 and < CIN1, resulting in two LBC specific, optimized assay reading software versions. The final dataset was analyzed using the ThinPrep and SurePath specific assay reading software versions.

### MGP-PCR and HPV typing using Luminex

All samples were HPV genotyped using MGP-PCR, primer targeting L1, and type-specific probes using Luminex detection technology, as previously described [34–36]. Briefly, 5 μL aliquots of extracted DNA were used in the MGP-PCR in a total volume of 25 μL. Forty-two beads, 37 different HPV-types, three HPV variants, and two ‘universal’ HPV probes, were included in the Luminex assay. Samples with a grey-zone result were retested in duplicate and HPV type(s) that were reproducible were considered definitive. All MGP-PCR and Luminex testing was performed at the Karolinska Institute, Stockholm, Sweden. MGP-PCR and HPV typing using Luminex was performed with the same protocol for both SurePath and ThinPrep collected samples.

### Cytology

#### SurePath procedure (Denmark)

Cytology was read following the Bethesda 2001 criteria. Hvidovre Hospital employs computer assisted screening using FocalPoint™ GS imaging system and SlideWizard™ (BD diagnostics, Burlington, NC), prior to cyto-screener review. HPV testing was performed after cytology evaluation; hence the cyto-screener was blinded to the HPV result upon evaluation, except for ASCUS cases which are reflex tested, routinely for HPV in accordance with current Danish National screening guidelines. All abnormal cytology findings were routinely adjudicated by a pathologist. According to National guidelines, women with LSIL were invited for repeat cytology testing after 6 months. Women with normal cytology were returned to routine screening after 3 years if aged 23–49 or 5 years if aged 50–59. All women included in the study were managed according to the routine guidelines for the Danish cervical cancer screening program.

#### ThinPrep procedure (Sweden)

Cytology was read following the Bethesda 2001 criteria. Manual screening review was performed by especially trained cyto-diagnosticians, with ambiguous cases resolved by specialist cytologist review. The Karolinska University Laboratory is the central cervical screening diagnostic laboratory for the Stockholm region, the capital region of Sweden. Within the cervical screening program, a randomized health services study was performed during 2012–2016 [37] for women aged 30–60. Half of the population was randomized to primary cytology, and half to primary HPV-based screening. In the cytology arm, ASCUS cases were routinely tested for HPV in accordance with guidelines. In the HPV arm, HPV-positive samples were tested with reflex cytology. In 2015, Sweden issued new guidelines for cervical screening recommending HPV-based screening for all women 30–64 years of age [38]. The Stockholm-Gotland region has been biobanking residuals from screening samples gradually since 2011 and all cervical screening samples since 2013 at the Clinical Cytology Biobank, Karolinska University Laboratory, Karolinska University Hospital.

### Histology

#### Danish procedures

In Denmark, women ≥30 years with ASCUS and a concurrent HPV-positive test result are referred to colposcopy with biopsies, as are women with high-grade squamous intraepithelial lesions (HSIL), atypical squamous cells – cannot exclude HSIL (ASC-H), atypical glandular cells (AGS) or cytological sign of carcinoma and women with continued ASCUS and LSIL cytological diagnosis. Danish screening guidelines requires biopsies from all aceto-white lesions or 4-counter clockwise random biopsies from all four quadrants in cases where no lesions are visible upon colposcopy. All histological data included in the study were retrieved from the Danish Pathology Data Register.

#### Swedish procedures

In Sweden, women are referred to colposcopy with biopsies according to similar guidelines as listed above for Denmark. For women with suspected high-grade disease, biopsies from lesions or random biopsies in the similar fashion should be performed. All histological data included in the study were retrieved from the Swedish National Cervical Screening Register.

### Data analysis

For CLART4S HPV, a sample was considered positive if at least one of the 14 genotypes (16, 18, 31,33, 35, 39, 45, 51, 52, 56, 58, 59, 66, & 68) was detected. HPV6, and/or HPV11 present alone without any of the other 14 HPV genotypes were considered HPV screen negative. The same was true for MGP-PCR. The CLART4S assay automatically reports genotype findings detected in an “uncertainty” range, if the visualization outcome falls close to the manufacturer cut-off. Reflecting routine practice at our facility, these genotypes are considered positive only if part of a multiple infection.

Clinical specificity and sensitivity values for CLART4S were compared to those of MGP-PCR using the non-inferior score test, where non-inferiority is defined as a relative specificity for <CIN2 of ≥98% and a relative sensitivity for ≥CIN2 of ≥90%. For the intra-laboratory reproducibility and inter-laboratory agreement, a lower confidence bound of ≥87% was used as a threshold [4]. The non-inferior score excel sheet was provided by VU University, Amsterdam, The Netherlands [4]. In the Swedish study population, Fisher’s exact test of homogeneity was applied to test homogeneity of distribution of HPV-status in women of < 30, and ≥ 30 years, respectively. For other statistical computations incl. 95% CI, the SPSS statistics 22 software was used.

## Results

### Clinical specificity and sensitivity

**Fig. 1:**
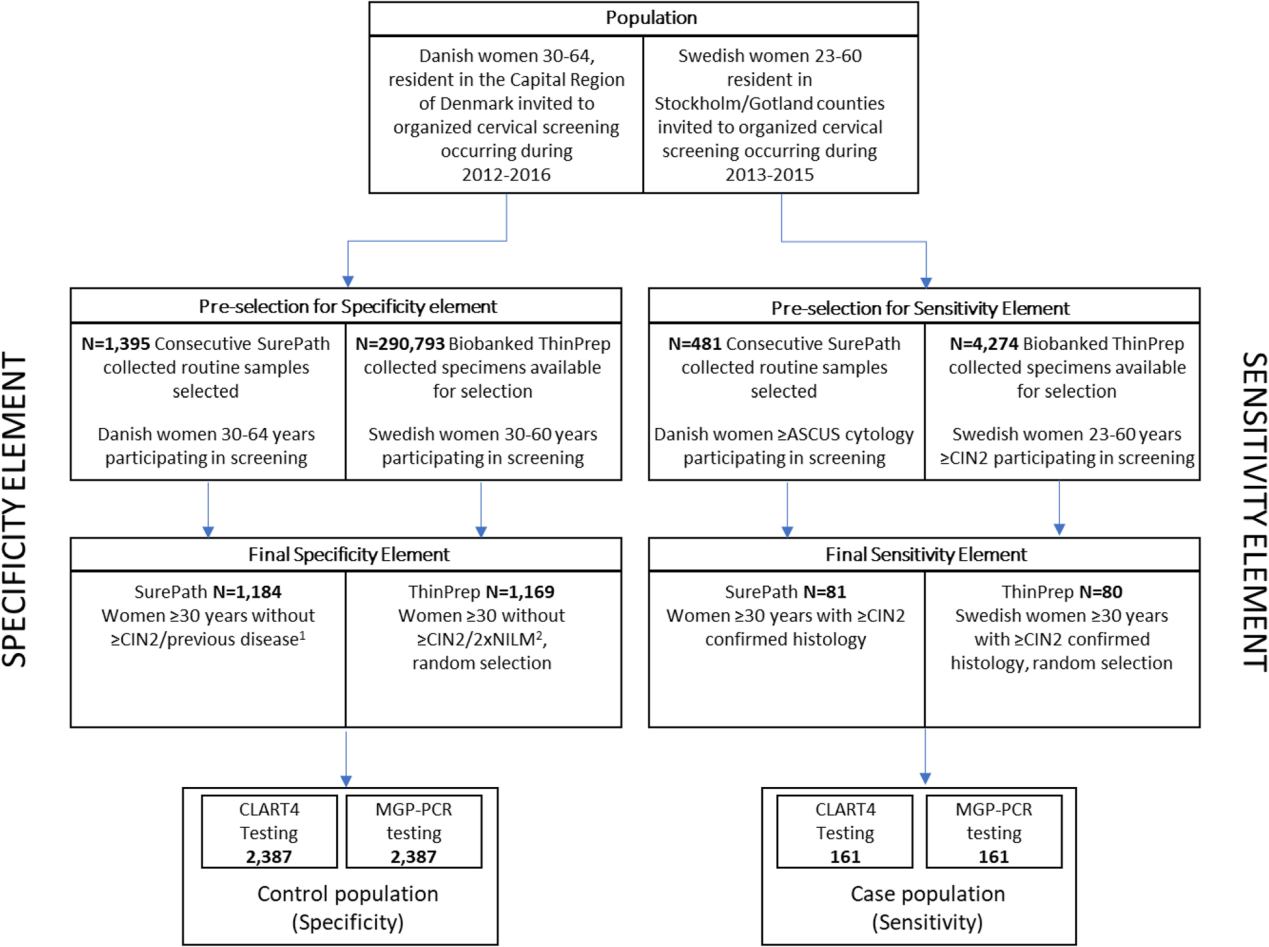
Flow chart over SurePath and ThinPrep sample collection for clinical validation 1: Previous disease defined as; a women with previous cytological diagnosis of ASCUS within the past 15 months, or a cytological diagnosis of more than ASCUS (>ASCUS) in the past 12 months, or previous cervical cancer or CIN in the previous 3 years, or insufficient/incomplete histological follow-up in the Danish register or diagnosis of ≥CIN2 follow-up after baseline analysis. 2: 2xNILM (Negative for Intraepithelial Lesions or Malignancy): Women with both the current and the previous cytology classified as normal ≥ASCUS: Atypical squamous cells of undetermined significance or above. ≥CIN2: Cervikal intraepitelial neoplasi 2 or above

For the specificity evaluation, 1184 SurePath screening samples from women ≥30 years (mean age 43.4, range 30–65) with <CIN2 histological follow-up were collected (Fig. 1). The specificity of 0.91 (95% CI: 0.89–0.92) of CLART4S was similar to MGP-PCR (0.91; 95% CI: 0.89–0.93, Table 1). The specificity of CLART4S was non-inferior to MGP-PCR (*p* = 0.01, Table 2).

**Table 1:** Clinical performance of CLART4S and MGP-PCR in SurePath and ThinPrep cervical cancer screening samples

Specificity and Sensitivity (95% Confidence Interval)				
	SurePath		ThinPrep	
Accuracy parameter	CLART4S	MGP-PCR	CLART4S	MGP-PCR
Specificity <CIN2	1074/1184 (0.91; 0.89–0.92)	1078/1184 (0.91; 0.89–0.93)	1096/1169 (0.94; 0.92–0.95)	1013/1169 (0.87; 0.85–0.89)
Relative Specificity	1.0 (0.97–1.02)	1.0	1.1 (1.05–1.11)	1.0
Sensitivity ≥CIN2	73/81 (0.90; 0.81–0.96)	75/81 (0.93; 0.85–0.97)	78/80 (0.98; 0.91–1.00)	80/80 (1.00; 0.95–1.00)
Relative sensitivity	0.97 (0.89–1.07)	1.0	0.98 (0.94–1.01)	1.0

**Table 2:** Genomica CLART4S findings among control screening samples without ≥CIN2 follow-up and case samples with confirmed ≥CIN2 histology collected in SurePath and ThinPrep, respectively, with MGP-PCR as comparator assay

	MGP-PCR			Non-inferior test
CLART4S	HPV pos^a^	HPV neg	Total	Statistics
Control (<CIN2)^b^				
SurePath				
HPV pos^a^	84	26	110	*P* = 0.008
HPV neg	22	1052	1074	
Total	106	1078	1184	
ThinPrep				
HPV pos	69	4	73	*P* = 0.000
HPV neg	87	1009	1096	
Total	156	1013	1169	
Case (≥CIN2)^c^				
SurePath				
Hr-HPV pos	71	4	75	*P* = 0.002
Hr-HPV neg	2	4	6	
Total	73	8	81	
ThinPrep				
Hr-HPV pos	78	0	78	*P* = 0.013
Hr-HPV neg	2	0	2	
Total	80	0	80	

^a^HPV positive for 16, 18, 31, 33, 35, 39, 45, 51, 52, 56, 58, 59, 66 and 68, ^b^Control population defined as women with 2 x NILM and no confirmed CIN2 histology, ^c^Case group defined as women with confirmed CIN2 or more histology

For specificity evaluation in ThinPrep samples, 1169 cervical cancer ThinPrep screening samples from women ≥30 years (mean age 38.3, range 30–63) with <CIN2 histological follow-up were collected. The specificity of CLART4S in ThinPrep samples was 0.94 (95% CI: 0.92–0.95), compared to 0.87 for MGP-PCR (95% CI 0.85–0.89, Table 1). The clinical specificity of CLART4S was non-inferior to that of MGP-PCR (*p* = 0.00, Table 2).

For sensitivity analysis, a total of 81 SurePath screening samples from women ≥30 years (mean 40.3, range 30–73) with confirmed ≥CIN2 histology were included (Table 1, Fig. 1). The case population included 21 women diagnosed with CIN2, 56 with CIN3 and four women with cervical cancer. The sensitivity of CLART4S was 0.90 (95% CI: 0.81–0.96) compared to 0.93 for MGP-PCR (95% CI: 0.85–0.97). The sensitivity of CLART4S was non-inferior to that of MGP-PCR (*p* = 0.002, Table 2). Four CIN3 cases were negative by CLART4S and positive by MGP-PCR (for HPV31, HPV68, HPV52 and HPV39/HPV68, respectively). One CIN2 and one CIN3 case were positive by CLART4S (HPV 31 and HPV 33, respectively) but negative by MGP-PCR.

For clinical validation of the CLART4S assay in ThinPrep samples, 21 ThinPrep cervical screening samples were collected from women < 30 and 59 were collected from women ≥30 years; all with histologically confirmed ≥CIN2 lesions during follow-up (mean age: 34.0, range 23–60). Of these, 51 was CIN2, 27 CIN3, 1 AIS and 1 cancer. There was no significant heterogeneity between samples from women above and below age 30 (*p* = 0.46). The sensitivity of CLART4 in ThinPrep samples was 0.98 (95% CI: 0.91–1.0), compared to 1.0 for MGP-PCR (95% CI: 0.95–1.00). The sensitivity of CLART4S in ThinPrep samples was non-inferior to MGP-PCR (*p* = 0.01, Table 2). Two CIN2 cases were negative by CLART4S and positive by MGP-PCR (HPV59 and HPV66, respectively).

### Comparison between CLART4S and MGP-PCR at genotype level

Table 3 (top) shows the genotypes detected comparing CLART4S and MGP-PCR on 1265 SurePath samples from the case and control populations combined. The hrHPV agreement between CLART4S and MGP-PCR was excellent (kappa: 0.83), also when investigated for 12 HPV individual genotypes (kappa good to excellent, range 0.60–0.95), but fair to moderate for genotypes HPV66 and HPV68 (kappa: 0.47 and 0.27, respectively, Table 3). McNemar test indicated statistically significant differences for HPV45 and HPV66 (Table 3), where CLART4S detected less infections of both genotypes.

**Table 3:** HPV Genotype distribution in 1265 SurePath screening samples and 1249 ThinPrep screening samples tested with Genomica CLART4S and MGP-PCR

HPV genotypes	Prevalence			Agreement (CLART4S/MGP-PCR)					Kappa	McNemar
	CLART4S	MGP-PCR	Ratio	+/+	+/−	−/+	−/−	Agreement		
SurePath^a^										
16	41	44	0.93	40	1	4	1220	99.6	0.94	0.38
18	10	11	0.91	9	1	2	1253	99.8	0.86	1.00
31	33	32	1.03	31	2	1	1231	99.8	0.95	1.00
33	11	7	1.57	7	4	0	1254	99.7	0.78	0.13
35	7	6	1.17	5	1	2	1257	99.8	0.77	1.00
39	20	13	1.54	10	10	3	1242	99.0	0.60	0.09
45	20	27	0.74	19	1	8	1237	99.3	0.81	0.04
51	10	7	1.43	6	4	1	1254	99.6	0.70	0.34
52	24	24	1.00	20	4	4	1237	99.4	0.83	1.00
56	12	11	1.09	10	1	2	1252	99.8	0.87	1.00
58	15	7	2.14	7	8	0	1250	99.4	0.63	0.01
59	10	11	0.91	8	2	3	1252	99.6	0.76	1.00
66	5	12	0.42	4	1	8	1252	99.3	0.47	0.04
68	14	8	1.75	3	11	5	1246	98.7	0.27	0.21
14 Hr-HPV	185	181	1.02	156	29	25	1055	95.7	0.83	0.68
ThinPrep^b^										
16	47	82	0.57	47	0	35	1167	96.0	0.72	0.00
18	9	12	0.75	9	0	3	1237	98.5	0.86	0.05
31	29	40	0.73	29	0	11	1209	97.9	0.84	0.00
33	20	18	1.11	17	3	1	1228	98.4	0.89	0.63
35	8	8	1.00	6	2	2	1239	98.4	0.75	1.00
39	3	9	0.33	3	0	6	1240	98,3	0.50	0.03
45	9	16	0.56	9	0	7	1233	98.2	0.69	0.02
51	14	23	0.61	13	1	10	1225	97.9	0.70	0.01
52	12	25	0.48	12	0	13	1224	97.7	0.64	0.00
56	4	8	0.50	4	0	4	1241	98.4	0.67	0.13
58	12	15	0.80	11	1	4	1233	98.3	0.81	0.38
59	2	10	0.20	2	0	8	1239	98.1	0.33	0.01
66	0	15	0.00	0	0	15	1234	97.5	0.00	0.00
68	9	10	0.90	4	5	6	1234	97.9	0.42	1.00
14 Hr-HPV	151	236	0.64	147	4	89	1009	91.4	0.72	0.00

^a^Data for both Control (1184) and Case (81) SurePath screening samples. In total 1265 SurePath samples

^b^Dato for both Control (1169) and Case (80) ThinPrep screening samples. In total 1249 ThinPrep samples

Table 3 (bottom) also shows the genotype distribution in 1249 ThinPrep samples from the case and control population combined. The overall HPV agreement was good (kappa 0.72), also for 10 individual HPV genotypes (kappa good to excellent, range 0.64–0.89), however poor to moderate for HPV39, 59, 66, 68 (kappa 0.5, 0.33, 0.0, 0.42, respectively). McNemar test indicated statistically significance for HPV 16, 31, 39, 45, 51, 52, 59 and HPV66.

### Intra-laboratory reproducibility and inter-laboratory agreement

The intra-laboratory reproducibility on SurePath samples was 95% (lower confidence bound: 0.93, kappa value: 0.87, Table 4). The inter-laboratory agreement on SurePath was 89% (lower confidence bound: 0.87, kappa value: 0.69). The reproducibility of the individual genotype results showed overall moderate to excellent agreement (range 0.50 to 1.00) for the intra-laboratory reproducibility (Table 5). For the inter-laboratory agreement, the genotype concordance was slightly lower with poor to excellent agreement (range 0.14 to 0.87, Table 5).

**Table 4:** Intra-laboratory reproducibility and inter-laboratory agreement of the CLART4S assay using SurePath screening samples

Assessment and site	HPV status	Copenhagen Laboratory Result 1		Total	Kappa	Lower bound
		Hr-HPV positive	Hr-HPV negative			
Intra-laboratory Reproducibility						
Copenhagen laboratory result 2	Hr-HPV positive	125	13	138	0.87	0.93
	Hr-HPV negative	14	388	402		
	Total	139	401	540		
Inter-laboratory Agreement						
Edinburgh Laboratory result	Hr-HPV positive	88	6	94	0.69	0.87
	Hr-HPV negative	51	395	446		
	Total	139	401	540

**Table 5:** Intra-laboratory reproducibility and inter-laboratory agreement of individual genotype findings in SurePath screening samples

Assessment and Hr-HPV type	No. of genotype findings per run or laboratory			No. negative for both runs	Kappa Value	95% Confidence Interval	
	Combined results	First run	Second run			Lower	Upper
Intra-laboratory reproducibility							
16	20	21	21	518	0.95	0.88	1.00
18	3	4	3	536	0.86	0.58	1.00
31	17	20	18	519	0.89	0.79	1.00
33	4	5	5	534	0.80	0.52	1.00
35	5	5	8	532	0.77	0.51	1.00
39	12	14	15	523	0.82	0.67	0.98
45	18	19	19	520	0.95	0.87	1.00
51	7	11	8	528	0.73	0.51	0.96
52	15	15	18	522	0.91	0.80	1.00
56	12	12	12	528	1.00	1.00	1.00
58	8	9	9	530	0.89	0.73	1.00
59	9	9	11	529	0.90	0.76	1.00
66	7	7	11	529	0.77	0.56	0.99
68	1	3	1	537	0.50	−0.1	1.00
Inter-laboratory agreement							
16	17	21	18	518	0.87	0.75	0.98
18	3	4	4	536	0.86	0.58	1.00
31	15	20	16	519	0.83	0.69	0.96
33	4	5	5	534	0.80	0.52	1.00
35	5	5	7	533	0.83	0.60	1.00
39	5	14	7	524	0.47	0.20	0.73
45	13	19	13	520	0.78	0.62	0.94
51	1	11	3	527	0.14	−0.11	0.38
52	9	15	9	525	0.75	0.55	0.94
56	3	12	4	527	0.37	0.07	0.67
58	5	9	5	531	0.71	0.44	0.98
59	3	9	4	530	0.46	0.12	0.80
66	3	7	5	531	0.50	0.15	0.84
68	1	3	2	536	0.40	−0.15	0.94

The intra-laboratory reproducibility in ThinPrep samples was 92% (lower confidence bound 0.90, kappa value 0.70, Table 6). The inter-laboratory agreement was 95% (lower confidence bound 0.93, kappa value 0.81). The reproducibility of the individual genotype result was overall good for inter and intra-laboratory agreement, but with poor to excellent agreements observed dependent upon genotype assessed (range 0.00–0.92 and 0.00–1.00, respectively, Table 7).

**Table 6:** Intra-laboratory reproducibility and inter-laboratory agreement of the CLART4S assay using ThinPrep screening samples

Assessment and site	HPV status	Stockholm Laboratory Result 1		Total	Kappa	Lower bound
		Hr-HPV positive	Hr-HPV negative			
Intra-laboratory Reproducibility						
Stockholm laboratory result 2	Hr-HPV positive	56	4	60	0.70	0.90
	Hr-HPV negative	34	397	431		
	Total	90	401	491		
Inter-laboratory Agreement						
Edinburgh Laboratory result	Hr-HPV positive	73	10	83	0.81	0.93
	Hr-HPV negative	17	395	412		
	Total	90	405	495

**Table 7:** Intra-laboratory reproducibility and inter-laboratory agreement of individual genotype findings in ThinPrep screening samples

Assessment and Hr-HPV type	No. of genotype findings per run or laboratory			No. negative for both runs	Kappa Value	95% Confidence Interval	
	Combined results	First run	Second run			Lower	Upper
Intra-laboratory reproducibility							
16	16	26	17	464	0.73	0.58	0.88
18	4	6	6	483	0.66	0.35	0.97
31	9	11	9	480	0.90	0.76	1.00
33	3	3	3	488	1.00	1.00	1.00
35	3	3	5	486	0.75	0.41	1.00
39	1	7	1	484	0.25	−0.15	0.64
45	6	8	7	482	0.80	0.57	1.00
51	2	8	2	483	0.40	0.01	0.78
52	8	11	8	480	0.84	0.66	1.00
56	0	8	0	483	0.00	0.00	0.00
58	4	8	5	482	0.61	0.30	0.92
59	0	1	0	490	0.00	0.00	0.00
66	0	0	0	491	0.00	0.00	0.00
68	4	7	4	484	0.72	0.43	1.00
Inter-laboratory agreement							
16	20	26	25	464	0.77	0.64	0.90
18	5	5	6	489	0.91	0.73	1.00
31	9	11	11	482	0.81	0.64	0.99
33	2	3	2	492	0.80	0.41	1.00
35	3	3	4	491	0.86	0.58	1.00
39	5	7	5	488	0.83	0.60	1.00
45	5	8	7	485	0.66	0.38	0.94
51	4	8	4	487	0.66	0.35	0.97
52	10	11	11	483	0.91	0.78	1.00
56	4	8	5	486	0.61	0.30	0.92
58	6	8	8	485	0.75	0.51	0.99
59	2	2	4	491	0.67	0.23	1.00
66	0	0	0	495	0.00	0.00	0.00
68	6	6	7	488	0.92	0.77	1.00

## Discussion

In this study, we validate in parallel the clinical performance on SurePath and ThinPrep-collected samples of the novel CLART4S assay to the comparator assay MGP-PCR. CLART4S is a full genotyping assay that detects 16 HPV genotypes individually. Here, the CLART4S assay was shown to have a similar clinical specificity and sensitivity performance to the comparator MGP-PCR assay for both SurePath and ThinPrep collected cervical cancer screening samples (Tables 1 and 2). The specificity (SurePath 0.01; ThinPrep 0.0) and sensitivity (SurePath 0.002; ThinPrep 0.01) of CLART4S were non-inferior to that of MGP-PCR for both LBC collection media. The Swedish case population contained 21 samples from women < 30 years of age which is not strictly in compliance with the international criteria. Fisher’s exact test of homogeneity was applied to test homogeneity of distribution of HPV-status in women of < 30, and ≥ 30 years, and found it to be similar. In consequence, the outcomes showed excellent sensitivity of CLART4 in both populations below, and above 30 years of age.

As comparator assay we used the MGP assay with Luminex which is a multiprimer system detecting at least 14 screening relevant HPV types [36]. Performance of the MGP assay showed a slightly higher sensitivity for detection of individual genotypes compared to the classical GP5+/6+ single primer pair.

At the level of overall HR-HPV detection, the CLART4S displayed intra-laboratory reproducibility and inter-laboratory agreement on SurePath collected samples within the recommended lower confidence bound of 87% (93 and 87%, respectively). However, the inter-laboratory reproducibility was borderline to acceptance which can cause quality assurance issues in non-expert laboratories. For ThinPrep, intra-laboratory reproducibility and inter-laboratory agreement was 90 and 93%, respectively (lower confidence bound, kappa: 0.70 and 0.81).

At the individual genotype level for SurePath samples, kappa values ranging 0.14–1.0 was observed displaying large variation in the CLART4s performance dependent upon genotype in question. Similar results were observed for ThinPrep collected samples (range 0 to 1.00).

The individual genotype concordance for the combined 1265 SurePath samples, showed good agreement between CLART4S and the comparator assay, except for HPV66 and HPV68. For the combined 1249 ThinPrep samples genotypic concordance was good, except for genotypes HPV39, HPV59, HPV66 and HPV68. According to the IARC classification [25] HPV66 is considered a *possible oncogenic* and HPV68 is considered to be a *probably oncogenic.* Neither are frequent in cervical cancers, nor are HPV59 and 39 despite their firm inclusion in the IARC high risk oncogenic group, so overall the clinical implication of a poor concordance may be marginal.

PapilloCheck (Greiner Bio-One) is the another full genotyping assay validated by the international guidelines [14], and the EUROarray (EUROIMMUN, Germany) [39], InnoLipa Extra II (Fujirebio, Belgium) [40] and Linear Array (Roche, CA) [41] assays has been validated in VALGENT3 against HC2. For PapilloCheck, the authors observed low concordance between PapilloCheck and GP5+/6+ for HPV68 with a kappa value of 0.00 [14]. For the remaining 13 genotypes, the concordance between PapilloCheck and GP5+/6+ showed kappa values from 0.62 to 0.95 [14]. Similarly, comparing Linear Array to InnoLipa, the authors found a kappa value of 0.345 for HPV68 [41].

With respect to validation of HPV assays, reproducibility of individually assay reported genotypes are rarely reported, nor required by the acceptance criteria. Reproducibility is measured only at the level of “presence/absence” of oncogenic HPV, yet this could mask substantial performance issues at the individual genotype level. To this end, a review or an update of the international criteria that incorporates an adjudication of type specific performance is timely. Arguably, this is particularly important given the increasing number of tests that have typing capability and the increasing interest in risk stratification using typing information. Another aspect that merits international discussion is how to utilize genotyping in clinical screening algorithms. A multitude of questions however remain as to how the clinical algorithms would change contingent to a result of the full typing, i.e. could women be referred for colposcopy versus re-test based upon specific risk estimates of single and multiple genotype combinations, or could full genotyping help improve screening by allowing extended follow-up for women with the lowest risk genotypes?

In this study, the sensitivity and specificity of CLART4S were slightly different between the ThinPrep and SurePath cohorts. Both collection media allow for additional testing for HPV prior to (primary screening) or subsequent to cytological evaluation (triage). Nevertheless, we argue that a substantial part of the observed assay performance difference stems from the LBC collection media. Firstly, the cellularity is not the same between the two sample collection methods, yet for CLART4S as well as all HPV screening assays, the analytical input per test has the same volume irrespectively of the sample collection media.

The SurePath vial contains 10 ml medium and the brush is left in the vial after sampling and prior to the cytology procedure. The ThinPrep vial contains 20 ml medium and the brush is rinsed in the medium and subsequently discarded. Moreover, SurePath contains a low concentration of formaldehyde added to the alcohol fixative to ensure adequate preservation of the cell material whereas ThinPrep uses methanol as the sole fixative. The difference in fixative has been a source of discussions internationally, with the claim that HPV analysis on SurePath samples can be challenged by the cross-linking between DNA and proteins driven by the formaldehyde content [42, 43]. However, correct preanalytical treatment of samples counters the impact of formaldehyde [44]. Furthermore, studies with BD Onclarity [16, 17], Hologic Aptima [45, 46], Roche cobas [47, 48] and Genomica CLART HPV2 [49] have previously shown that SurePath collected samples can safely be used for HPV analysis. ThinPrep collected LBC medium on the other hand does not contain formaldehyde and consequently is considered less challenging with respect to HPV testing.

Finally, we do take note that SurePath and ThinPrep population were collected from two different cervical cancer screening programs, which could also contribute to the assay performance variance observed. From an operational perspective, an outcome of this study has been that the manufacturer of CLART4s equipped two software versions on the HPV analysis platform, optimized for either SurePath and ThinPrep collected samples, respectively, which constitute an adept solution to the issue.

## Conclusion

In conclusion, our data shows that the CLART4S assay is equivalent and non-inferior to the comparator MGP-PCR assay with respect to clinical sensitivity and specificity, for both SurePath and ThinPrep collected cervical cancer screening samples. Moreover, inter-laboratory reproducibility and intra-laboratory agreement fulfill the international validation criteria for both media types. Given the full genotyping capabilities of the assay, the CLART4S is a suitable candidate for future primary HPV screening. Finally, we would put forth two suggestions: 1) That all HPV assays are validated on both ThinPrep and SurePath medias rather than to assume similar performance across both; 2) That type-specific validation metrics become part of validation criterias to accommodate the evolution of HPV assays towards individual genotype detection.

## Data Availability

The datasets generated and/or analysed during the current study are not publicly available as per compliance with the current interpretation of the EU-General Data Protection Regulation. Data are available for 3rd party on reasonable request to the corresponding author and after the establishment of EU-GDPR data-handler agreement between parties

## Abbreviations

HPV: Human Papillomavirus
HC2: Hybrid Capture 2
LBC: Liquid Based Cytology
CIN: Cervical Intraepithelial Neoplasia
ASCUS: Atypical squamous cells of undetermined significance
LSIL: Low-grade Squamous Intraepithelial Lesion
CLART4S: CLART® HPV4S
MGP-PCR: Modified General Primers GP5+/6+
HSIL: High-grade Squamous Intraepithelial Lesion
ASC-H: Atypical squamous cells- cannot exclude HSIL
AIS: Adenocarcinoma In Situ

## References

[1] Arbyn M, Ronco G, Anttila A, Meijer CJ, Poljak M, Ogilvie G, Koliopoulos G, Naucler P, Sankaranarayanan R, Peto J (2012). Evidence regarding human papillomavirus testing in secondary prevention of cervical cancer. Vaccine.

[2] Ronco G, Dillner J, Elfstrom KM, Tunesi S, Snijders PJ, Arbyn M, Kitchener H, Segnan N, Gilham C, Giorgi-Rossi P, Berkhof J, Peto J, Meijer CJ, International HPVswg (2014). Efficacy of HPV-based screening for prevention of invasive cervical cancer: follow-up of four European randomised controlled trials. Lancet.

[3] Poljak M, Kocjan BJ, Ostrbenk A, Seme K (2016). Commercially available molecular tests for human papillomaviruses (HPV): 2015 update. J Clin Virol.

[4] Meijer CJ, Berkhof J, Castle PE, Hesselink AT, Franco EL, Ronco G, Arbyn M, Bosch FX, Cuzick J, Dillner J, Heideman DA, Snijders PJ (2009). Guidelines for human papillomavirus DNA test requirements for primary cervical cancer screening in women 30 years and older. Int J Cancer.

[5] Arbyn M, Snijders PJ, Meijer CJ, Berkhof J, Cuschieri K, Kocjan BJ, Poljak M (2015). Which high-risk HPV assays fulfil criteria for use in primary cervical cancer screening?. Clin Microbiol Infect.

[6] Boers A, Wang R, Slagter-Menkema L, van Hemel BM, Ghyssaert H, van der Zee AG, Wisman GB, Schuuring E (2014). Clinical validation of the Cervista HPV HR test according to the international guidelines for human papillomavirus test requirements for cervical cancer screening. J Clin Microbiol.

[7] Depuydt CE, Benoy IH, Beert JF, Criel AM, Bogers JJ, Arbyn M (2012). Clinical validation of a type-specific real-time quantitative human papillomavirus PCR against the performance of hybrid capture 2 for the purpose of cervical cancer screening. J Clin Microbiol.

[8] Heideman DA, Hesselink AT, Berkhof J, van Kemenade F, Melchers WJ, Daalmeijer NF, Verkuijten M, Meijer CJ, Snijders PJ (2011). Clinical validation of the cobas 4800 HPV test for cervical screening purposes. J Clin Microbiol.

[9] Heideman DA, Hesselink AT, van Kemenade FJ, Iftner T, Berkhof J, Topal F, Agard D, Meijer CJ, Snijders PJ (2013). The Aptima HPV assay fulfills the cross-sectional clinical and reproducibility criteria of international guidelines for human papillomavirus test requirements for cervical screening. J Clin Microbiol.

[10] Hesselink AT, Berkhof J, van der Salm ML, van Splunter AP, Geelen TH, van Kemenade FJ, Bleeker MG, Heideman DA (2014). Clinical validation of the HPV-risk assay, a novel real-time PCR assay for detection of high-risk human papillomavirus DNA by targeting the E7 region. J Clin Microbiol.

[11] Hesselink AT, Meijer CJ, Poljak M, Berkhof J, van Kemenade FJ, van der Salm ML, Bogaarts M, Snijders PJ, Heideman DA (2013). Clinical validation of the Abbott RealTime high risk HPV assay according to the guidelines for human papillomavirus DNA test requirements for cervical screening. J Clin Microbiol.

[12] Hesselink AT, Sahli R, Berkhof J, Snijders PJ, van der Salm ML, Agard D, Bleeker MC, Heideman DA (2016). Clinical validation of Anyplex II HPV HR detection according to the guidelines for HPV test requirements for cervical cancer screening. J Clin Virol.

[13] Iacobellis M, Violante C, Notarachille G, Simone A, Scarfi R, Giuffre G (2018). Clinical validation of REALQUALITY RQ-HPV screen according to the international guidelines for human papillomavirus DNA test requirements for cervical screening. Virol J.

[14] Hesselink AT, Heideman DA, Berkhof J, Topal F, Pol RP, Meijer CJ, Snijders PJ (2010). Comparison of the clinical performance of PapilloCheck human papillomavirus detection with that of the GP5+/6+−PCR-enzyme immunoassay in population-based cervical screening. J Clin Microbiol.

[15] Ejegod D, Serrano I, Cuschieri KS, Nussbaumer WA, Vaughan LM, Ahmad AS, Cuzick J, Bonde J. Clinical validation of the BD Onclarity HPV assay using a non-inferiority test. Med Microbiol Diagnos. 2013;S3.

[16] Ejegod D, Bottari F, Pedersen H, Sandri MT, Bonde J (2016). The BD Onclarity HPV assay on samples collected in SurePath medium meets the international guidelines for human papillomavirus test requirements for cervical screening. J Clin Microbiol.

[17] Stoler MH, Wright TC, Parvu V, Vaughan L, Yanson K, Eckert K, Karchmer T, Kodsi S, Cooper CK (2018). The Onclarity human papillomavirus trial: design, methods, and baseline results. Gynecol Oncol.

[18] Arbyn M, Tommasino M, Depuydt C, Dillner J (2014). Are 20 human papillomavirus types causing cervical cancer?. J Pathol.

[19] Bonde J, Bottari F, Parvu V, Pedersen H, Yanson K, Iacobone AD, Kodsi S, Landoni F, Vaughan L, Ejegod DM, Sandri MT. Bayesian analysis of baseline risk of CIN2 and >/=CIN3 by HPV genotype in a European referral cohort. Int J Cancer. 2019. 10.1002/ijc.32291. 10.1002/ijc.32291 PMC6617734 30895602

[20] Bzhalava D, Guan P, Franceschi S, Dillner J, Clifford G (2013). A systematic review of the prevalence of mucosal and cutaneous human papillomavirus types. Virology.

[21] Khan MJ, Castle PE, Lorincz AT, Wacholder S, Sherman M, Scott DR, Rush BB, Glass AG, Schiffman M (2005). The elevated 10-year risk of cervical precancer and cancer in women with human papillomavirus (HPV) type 16 or 18 and the possible utility of type-specific HPV testing in clinical practice. J Natl Cancer Inst.

[22] Li N, Franceschi S, Howell-Jones R, Snijders PJ, Clifford GM (2011). Human papillomavirus type distribution in 30,848 invasive cervical cancers worldwide: variation by geographical region, histological type and year of publication. Int J Cancer.

[23] Bonde JH, Sandri MT, Gary DS, Andrews JC. Clinical utility of human papillomavirus genotyping in cervical Cancer Screening: A Systematic Review. J Low Genit Tract Dis. 2019. 10.1097/LGT.0000000000000494. 10.1097/LGT.0000000000000494 PMC6924950 31714325

[24] Serrano B, de Sanjose S, Tous S, Quiros B, Munoz N, Bosch X, Alemany L (2015). Human papillomavirus genotype attribution for HPVs 6, 11, 16, 18, 31, 33, 45, 52 and 58 in female anogenital lesions. Eur J Cancer.

[25] Group IMw (2007). IARCH monographs on the evaluation of Carconogenic risks of humans.

[26] Thomsen LT, Frederiksen K, Munk C, Junge J, Iftner T, Kjaer SK (2015). Long-term risk of cervical intraepithelial neoplasia grade 3 or worse according to high-risk human papillomavirus genotype and semi-quantitative viral load among 33,288 women with normal cervical cytology. Int J Cancer.

[27] Kjaer SK, Munk C, Junge J, Iftner T (2014). Carcinogenic HPV prevalence and age-specific type distribution in 40,382 women with normal cervical cytology, ASCUS/LSIL, HSIL, or cervical cancer: what is the potential for prevention?. Cancer Causes Control.

[28] Schiffman M, Hyun N, Raine-Bennett TR, Katki H, Fetterman B, Gage JC, Cheung LC, Befano B, Poitras N, Lorey T, Castle PE, Wentzensen N (2016). A cohort study of cervical screening using partial HPV typing and cytology triage. Int J Cancer.

[29] Smelov V, Elfstrom KM, Johansson AL, Eklund C, Naucler P, Arnheim-Dahlstrom L, Dillner J (2015). Long-term HPV type-specific risks of high-grade cervical intraepithelial lesions: a 14-year follow-up of a randomized primary HPV screening trial. Int J Cancer.

[30] Schiffman M, Vaughan LM, Raine-Bennett TR, Castle PE, Katki HA, Gage JC, Fetterman B, Befano B, Wentzensen N (2015). A study of HPV typing for the management of HPV-positive ASC-US cervical cytologic results. Gynecol Oncol.

[31] RIVM. 2017. Framework for the execution of cervical Cancer population screening, https://www.rivm.nl/en/Documents_and_publications/Professional_Serviceable/Guides/Disease_Prevention_and_Healthcare/cervical_cancerscreening/Framework_for_the_Execution_of_Cervical_Cancer_Population_Screening. National Institute for public health and the Enviroment, Ministry of Health, welfare and sport.

[32] Xu L, Benoy I, Cuschieri K, Poljak M, Bonde J, Arbyn M (2019). Accuracy of genotyping for HPV16 and 18 to triage women with low-grade squamous intraepithelial lesions: a pooled analysis of VALGENT studies. Expert Rev Mol Diagn.

[33] Arbyn M, Xu L, Verdoodt F, Cuzick J, Szarewski A, Belinson JL, Wentzensen N, Gage JC, Khan MJ (2017). Genotyping for human papillomavirus types 16 and 18 in women with minor cervical lesions: a systematic review and meta-analysis. Ann Intern Med.

[34] Lagheden C, Eklund C, Lamin H, Kleppe SN, Lei J, Elfstrom KM, Sundstrom K, Andrae B, Sparen P, Dillner J (2018). Nationwide comprehensive human papillomavirus (HPV) genotyping of invasive cervical cancer. Br J Cancer.

[35] Schmitt M, Bravo IG, Snijders PJ, Gissmann L, Pawlita M, Waterboer T (2006). Bead-based multiplex genotyping of human papillomaviruses. J Clin Microbiol.

[36] Soderlund-Strand A, Carlson J, Dillner J (2009). Modified general primer PCR system for sensitive detection of multiple types of oncogenic human papillomavirus. J Clin Microbiol.

[37] Lamin H, Eklund C, Elfstrom KM, Carlsten-Thor A, Hortlund M, Elfgren K, Tornberg S, Dillner J (2017). Randomised healthcare policy evaluation of organised primary human papillomavirus screening of women aged 56-60. BMJ Open.

[38] Socialstyrelsen. 2019. Livmoderhalscancer – screening med cytologi och HPV-test. https://www.socialstyrelsense/regler-och-riktlinjer/nationella-screeningprogram/slutliga-rekommendationer/livmoderhalscancer/.

[39] Viti J, Poljak M, Ostrbenk A, Bhatia R, Alcaniz Boada E, Cornall AM, Cuschieri K, Garland S, Xu L, Arbyn M (2018). Validation of EUROArray HPV test using the VALGENT framework. J Clin Virol.

[40] Xu L, Padalko E, Ostrbenk A, Poljak M, Arbyn M. Clinical evaluation of INNO-LiPA HPV genotyping EXTRA II assay using the VALGENT framework. Int J Mol Sci. 2018;19. 10.3390/ijms19092704 PMC6165258 30208597

[41] Xu L, Ostrbenk A, Poljak M, Arbyn M (2018). Assessment of the Roche linear Array HPV genotyping test within the VALGENT framework. J Clin Virol.

[42] Moelans CB, Oostenrijk D, Moons MJ, van Diest PJ (2011). Formaldehyde substitute fixatives: effects on nucleic acid preservation. J Clin Pathol.

[43] Moelans CB, ter Hoeve N, van Ginkel JW, ten Kate FJ, van Diest PJ (2011). Formaldehyde substitute fixatives. Analysis of macroscopy, morphologic analysis, and immunohistochemical analysis. Am J Clin Pathol.

[44] Agreda PM, Beitman GH, Gutierrez EC, Harris JM, Koch KR, LaViers WD, Leitch SV, Maus CE, McMillian RA, Nussbaumer WA, Palmer ML, Porter MJ, Richart GA, Schwab RJ, Vaughan LM (2013). Long-term stability of human genomic and human papillomavirus DNA stored in BD SurePath and Hologic PreservCyt liquid-based cytology media. J Clin Microbiol.

[45] Chernesky M, Jang D, Escott N, Gilchrist J, Li J, Elit L, Lytwyn A, Smieja M, Ratnam S, Arias M, Getman D, Weinbaum B, Kirkconnell B, Dockter J (2017). Detection of cervical precancerous lesions with Aptima HPV assays using SurePath preservative fluid specimens. Papillomavirus Res.

[46] Rebolj M, Preisler S, Ejegod DM, Bonde J, Rygaard C, Lynge E (2013). Prevalence of human papillomavirus infection in unselected SurePath samples using the APTIMA HPV mRNA assay. J Mol Diagn.

[47] Krevolin MD, Hardy D, Pane J, Aslam S, Behrens CM (2017). Development and validation of a Preanalytic procedure for performing the cobas HPV test in SurePath preservative fluid. J Mol Diagn.

[48] Preisler S, Rebolj M, Untermann A, Ejegod DM, Lynge E, Rygaard C, Bonde J (2013). Prevalence of human papillomavirus in 5,072 consecutive cervical SurePath samples evaluated with the Roche cobas HPV real-time PCR assay. PLoS One.

[49] Bonde J, Rebolj M, Ejegod DM, Preisler S, Lynge E, Rygaard C (2014). HPV prevalence and genotype distribution in a population-based split-sample study of well-screened women using CLART HPV2 human papillomavirus genotype microarray system. BMC Infect Dis.

